# DICED (Database of Identified Cleavage Sites Endemic to Diseases States): A Searchable Web Interface for Terminomics/Degradomics

**DOI:** 10.1002/pmic.202500007

**Published:** 2025-05-12

**Authors:** Jayadev Joshi, Sumit Bhutada, Daniel R. Martin, Joyce Guzowski, Daniel Blankenberg, Suneel S. Apte

**Affiliations:** ^1^ Center for Computational Life Sciences Cleveland Clinic Research Cleveland Ohio USA; ^2^ Department of Biomedical Engineering Cleveland Clinic Research Cleveland Ohio USA; ^3^ Central Administration Cleveland Clinic Research Cleveland Ohio USA

**Keywords:** data access, degradomics, proteases, proteolysis, proteomics, terminomics

## Abstract

Proteolysis is an irreversible posttranslational modification with immense biological impact. Owing to its high disease significance, there is growing interest in investigating proteolysis on the proteome scale, termed degradomics. We developed ‘Database of Identified Cleavage sites Endemic to Disease states’ (DICED; https://diced.lerner.ccf.org/), as a searchable knowledgebase to promote collaboration and knowledge sharing in degradomics. DICED was designed and constructed using Python, JavaScript, HTML, and PostgreSQL. Django (https://www.djangoproject.com) was chosen as the primary framework for its security features and support for agile development. DICED can be utilized on major web browsers and operating systems for easy access to high‐throughput mass spectrometry‐identified cleaved protein termini. The data was obtained using N‐terminomics, comprising N‐terminal protein labeling, labeled peptide enrichment, mass spectrometry and positional peptide annotation. The DICED database contains experimentally derived N‐terminomics peptide datasets from tissues, diseases, or digests of tissue protein libraries using individual proteases and is searchable using UniProt ID, protein name, gene symbol or up to 100 peptide sequences. The tabular output format can be exported as a CSV file. Although DICED presently accesses data from a single laboratory, it is freely available as a Galaxy tool and the underlying database is scalable, permitting addition of new datasets and features.

CSRF: cross‐site request forgery
ECM: extracellular matrix
SQL: structured query language
TAILS: terminal amine‐isotopic labeling of substrates
TMT: tandem mass tags
XSS: cross‐site scripting

1

Proteolysis is an irreversible posttranslational modification with a major role in protein degradation and is an activation mechanism for many proteins. Intracellularly, the proteasome, a large cellular protein complex for regulated degradation of cytosolic and nuclear proteins, has a crucial role in control of the cell cycle and other vital processes. Internalized membrane and secreted proteins are rapidly degraded within the acidic environment of lysosomes. Secreted proteins are cleaved by numerous secreted and cell‐surface proteases and extracellular matrix (ECM) is extensively modified by proteases as a requirement for its assembly and disassembly [1–4]. Systems level analysis of proteolysis in common diseases including osteoarthritis, infectious/inflammatory disorders, and cancer [5–9] has demonstrated that it occurs on an unexpectedly vast scale, which was not apparent from the prior use of candidate approaches limited to single substrates and proteases. Despite the broad significance of proteolysis, the proteolytic landscapes of healthy and diseased tissues remain relatively poorly defined. There is a pressing need both for the elucidation of these disease landscapes and dissemination of the knowledge obtained so that it can be effectively applied.

Degradomics refers to systems approaches that map proteolysis and protease activity. The resulting ensemble of proteolytic products and proteases constitutes a proteolytic landscape and is referred to as the degradome. Degradomics is greatly facilitated by N‐terminomics, a specialized branch of proteomics dedicated to identifying and analyzing protein N‐termini, including natural and proteolytically derived termini [10 ^,^ 11]. In contrast, C‐terminomics is more challenging, owing to the relative nonreactivity of protein C‐termini. However, defining either an N‐ or C‐terminus essentially identifies the same cleaved peptide bond. Terminomics strategies utilize LC‐MS/MS for high‐throughput and unbiased proteome‐wide coverage. Among several N‐terminomics strategies, terminal amine isotopic labeling of substrates (TAILS) [12, 13] has been widely used. TAILS permits single‐label, duplex, and multiplexed applications depending on the N‐terminal mass adduct used, e.g., dimethyl isotopes, isobaric tags for relative and absolute quantitation (iTRAQ) or tandem mass tags (TMT) (Kockmann, 2016). TAILS was previously used for forward degradomics (unbiased acquisition of all protein N‐termini in a sample) (e.g., [6, 14, 8, 15] as well as reverse degradomics (eliciting the degradome of a single protease after digestion of a protein substrate library, or by comparison of wild‐type and mutant cells or tissues) (e.g., [6, 7, 16]).

Among online tools and reference databases available for N‐terminomics, CLIPPER (https://github.com/UadKLab/CLIPPER‐2.0) [17] provides positional peptide annotation, statistical analysis of terminomics datasets, and data visualization. MEROPS (http://www.ebi.ac.uk/merops/) [18] is a curated database of experimentally verified substrates and cleavage sites of specific proteases from numerous organisms. TopFIND 4.1 integrates information from the UniProt knowledgebase (UniProtKB), MEROPS peptidase database, and experimental terminomics studies of eight organisms, whereas TopFINDer enables annotation of terminomics datasets [19, 20]. Here, we have developed an interface for simultaneous queries across multiple N‐terminomics datasets, embodying forward as well as reverse degradomics. The new resource facilitates in‐lab analysis across datasets as well as N‐terminomics data access by biologists, clinicians, and others who find themselves unable to handle large datasets downloaded from ProteomeXchange, the repository where terminomics datasets are deposited as a condition of publication.

As constructed (Figure 1), DICED provides an open‐source database accessible on common web browsers. Since hosting biomedical data, especially in hospital‐based research centers, presents data security concerns. DICED was developed using the Django framework, whose core focus is ensuring data security. This strength, combined with flexibility, ease of use, extensibility, and robustness, allowed rapid and secure development. PostgreSQL, an advanced open‐source relational database system with high reliability, scalability, and strong support for structured query language (SQL) standards was used as the database tool. The powerful indexing and querying capabilities of PostgreSQL enable efficient retrieval and management of complex proteomics data. We used Python as our programming language for back‐end operations due to its versatility, ease of use, and vast ecosystem of libraries and frameworks. Python offers a simple syntax that promotes readability and reduces development time, making it suitable for rapid prototyping and building applications. For front‐end operations primarily JavaScript, HTML, and CSS were used. We leveraged Django's built‐in tools, such as an admin interface and ORM (Object‐Relational Mapping), to streamline common development tasks and provide controlled access to various users based on their roles. DICED has three major components. The user interface (Figure 2) provides access to the data query system, data statistics, and versions. Since user feedback is crucial for continuously improving the interface and tools, an easy‐to‐use form is available for submitting error reports, comments, or suggestions.

**FIGURE 1:**
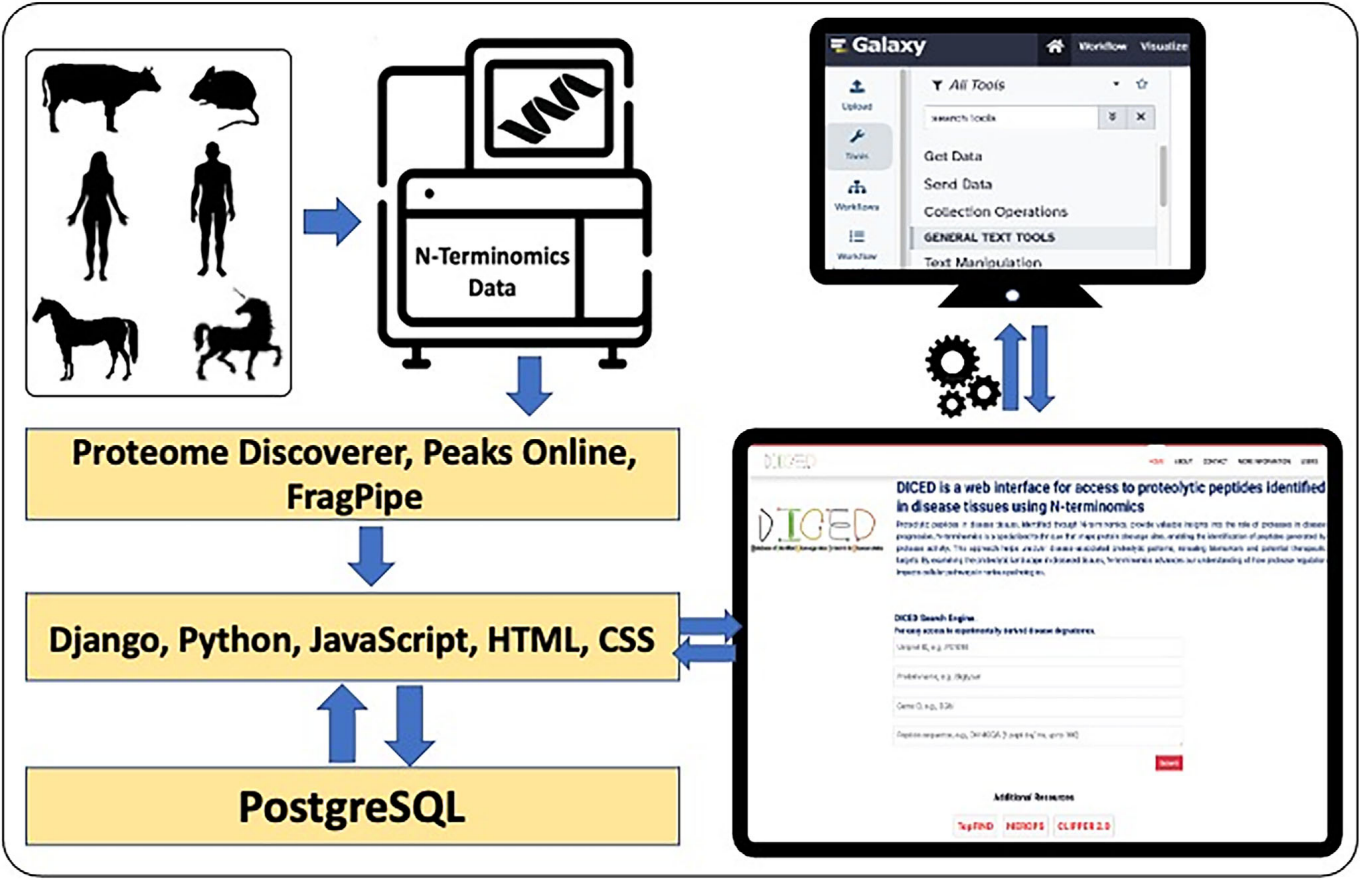
Overview of DICED architecture: The N‐terminomics data is obtained from distinct LC‐MS/MS instruments and analytic software as shown. DICED architecture is designed around an intuitive API‐based interface, built upon the Django web framework. A data source tool can fetch data directly within the Galaxy platform (see also Figures S1–S2).

**FIGURE 2:**
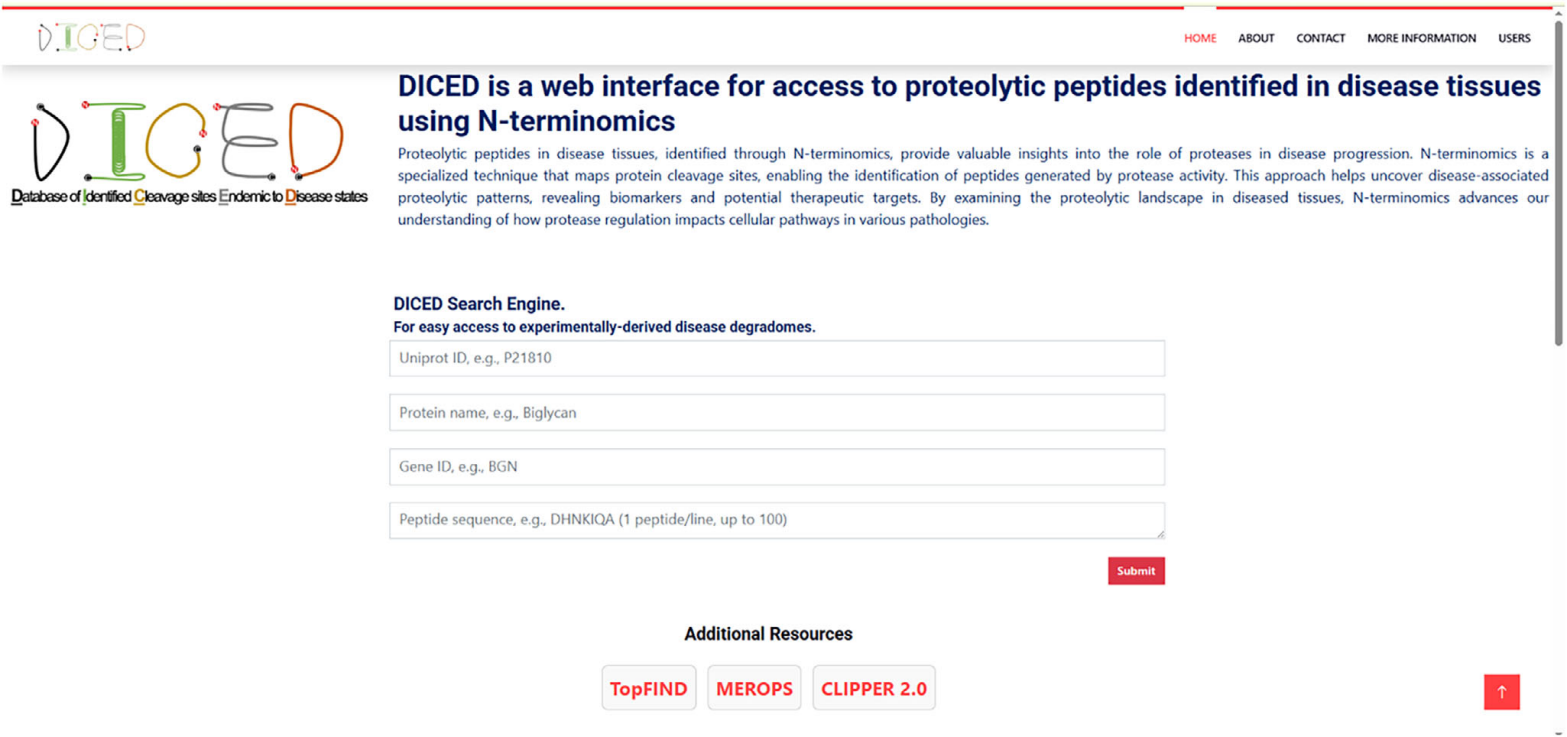
DICED home page: The search boxes for querying the DICED database are on the home page. UniProt ID, protein name, gene symbol, or 1–100 peptide sequence(s) can be submitted. DICED provides links to major online resources typically used for analysis and visualization of terminomics data.

The data was obtained from N‐terminomics experiments using dimethyl, iTRAQ, or TMT labels that were analyzed on high‐resolution mass spectrometers and analyzed with a false discovery rate of 0.01 using Proteome Discoverer (for Orbitrap instrument data), or FragPipe and Peaks Online (for Bruker timsTOF Pro 2 data) (Figure 1). The datasets accessed by DICED searches contain sequences of N‐terminally labeled peptides as well as peptides with nontryptic C‐termini observed in the data, since trypsin was used as the working protease. DICED provides searchable access to these peptide sequences without providing further specifics of physiological or proteolytic origin, attribution to specific proteases unless the peptide was specifically observed in one or more experimental reverse degradomics datasets, or alternatively splicing.

For versatile access, the search query can be a UniProt ID, protein name, gene symbol, or between 1 and 100 peptide sequences (Figure 2). The output is displayed in a table which can be downloaded as a CSV file (Figure 3, Figure S3). To ensure data integrity, reproducibility, and quality, we implemented automated database versioning. Database administrators (admin), can access an advanced Django admin user interface, e.g., to download the database backup file and upload new datasets. We customized the admin user interface for streamlining administrative tasks for enhanced data management capabilities to provide a robust toolset for data administration and management tasks.

**FIGURE 3:**
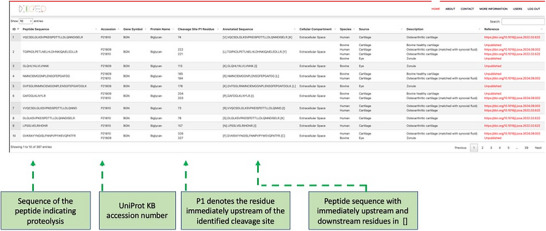
Sample data output file. An example of a specific search with “aggrecan” as the search term demonstrates key features of the output file. The green boxes provide additional annotation of specific columns.

Database upload is currently permitted only by designated site administrators. Uploads of additional terminomics data can be made in two ways: to upload a fresh file, the data file should be in tabular format, and for efficient data upload, resumable upload has been implemented. To ensure the quality and integrity of the data before uploading, a strict data validation process is in place which checks the data file formats, dataset column names, and data types before uploading the data file. In the second data uploading method, data can be uploaded using a database backup file which must be in .JSON file format and previously downloaded through the DICED interface from the backup download tab via the advanced user interface.

Django provides built‐in protections against common web vulnerabilities such as SQL injection, cross‐site scripting (XSS), cross‐site request forgery (CSRF), and clickjacking. We also utilize Django's built‐in secure user authentication and authorization mechanisms to grant privileged access to admin users. A user can only access the query page of the database while the accessibility of the advanced users is managed by an admin interface. DICED is designed on an intuitive API framework that allows users to extract data programmatically. This API‐based framework enables developers to integrate this database into their own applications. The database is hosted on a secure virtual machine within the Cleveland Clinic's computational facility to ensure the privacy and confidentiality of the proteomics data. The website is maintained by a team of experienced developers and researchers, ensuring the accuracy and reliability of the proteomics data. Advanced users can access all this information through the admin interface. We have developed a Galaxy data source tool that allows users to send queried data directly to the Galaxy interface for further analysis (Figure S1). Galaxy (www.usegalaxy.org) is a browser‐based data analysis platform that offers a user‐friendly interface for data analysis and importantly, numerous proteomics applications which can be used for further analysis of data output from DICED (Figure S2).

DICED presently accesses several experimental datasets from only the authors’ laboratory, including published forward degradomes [6–8], reverse degradomics for proteases HtrA1, MMP13, CMA1, and ADAMTS5 [6, 7], and unpublished forward degradomes. DICED is scalable to larger databases than presently available, permitting use as a future community resource.

Figures 3 andS3 show examples of the output from protein queries that illustrate the information that can be obtained. Depending on the search term and datasets available, the output can indicate peptides found across different disease states, different species, or if found in both forward and reverse degradomics experiments, attribute the cleavage observed in a forward degradome to a specific protease. If a proteolytic peptide has an identical sequence in two or more species, the sequence is appropriately annotated to reflect this (Figure S3).

DICED is not intended to duplicate the functionalities of TopFIND, CLIPPER 2.0, and MEROPS. TopFIND and CLIPPER remain exceptional tools for downstream analysis of large degradomics datasets including those searched in DICED to assist with the common goal of annotating every proteolytic event with its causal protease. Because forward degradomics of human disease is relatively new, especially when integrated with reverse degradomics for causal protease assignment [6, 7], annotation of disease by protease contributions is sparse and most proteolytic cleavages remain unattributed to cognate proteases. The magnitude of the knowledge gap is demonstrated by comparison of the large numbers of proteolytic peptides from 5 cartilage proteins identified by DICED which were absent in TopFIND or MEROPS (Figure S4).

Among additional resources recently available for terminomics/degradomcs, the R package Proteasy facilitates data extraction from MEROPS [21]. PeptideCutter (https://web.expasy.org/peptide_cutter/) predicts potential cleavage sites from a limited set of proteases with well‐defined specificity and returns the query sequence with the predicted cleavage sites mapped, but these sites have not necessarily been experimentally verified. However, few human proteases cleave their substrates with rigorous sequence specificity and thus, their cleavage sites must be determined experimentally, e.g., using TAILS. Included in this category, for example, are nearly all secreted and cell‐surface mammalian metalloproteinases, which constitute an important class for ECM remodeling [22]. CaspSites (www.caspsites.org) [23] is a free‐to‐use database and web application for experimentally observed human caspase substrates and cleavage sites identified using N‐terminomics, but is limited to a specific protease class.

Since the DICED database is not confined to a single species, protease, tissue, or disease, it will improve uncovering of protease activities across species, organs, and diseases. For example, searching for biglycan proteolytic peptides in DICED retrieved several proteolytic peptides from the human OA and bovine cartilage N‐terminomes and identified a cleavage site for MMP13 (Figure S3). The knowledge resource provided by DICED has several downstream applications. Peptide sequences accessed in DICED could be used to develop proteolytic profiles of diseases or for reporting the activity of specific proteases. Protein termini can be used to generate neo‐epitope antibodies which are widely used in research and clinical diagnostics [24–28]. Multiple/parallel reaction monitoring assays that include dozens to hundreds of selected proteolytic peptides representing the activity profiles of specific proteases could be used as disease biomarkers or to guide development of specific anti‐protease therapies, e.g., to define cross‐inhibition of other proteases or effectiveness of protease inhibition. Proteolytic peptides extracted from DICED could be used to query PeptideAtlas (https://peptideatlas.org/) [29] to determine which proteolytic peptides are found in the plasma, urine, or extracellular vesicle proteomes, and to annotate peptides within PeptideAtlas by potential tissue origin and as proteolytically generated. In the future, it is feasible that DICED or a future iteration could be upscaled for deposition of terminomics datasets from any laboratory according to specific submission guidelines or adopted for internal laboratory use. To facilitate this, we have made the DICED framework available as an open‐source project for local installation (GitHub project: https://github.com/jaidevjoshi83/DICEDWebApp.git).

## Conflicts of Interest

D.B. has a significant financial interest in GalaxyWorks, a company that may have a commercial interest in the results of this research and technology. This potential conflict of interest has been reviewed and is managed by the Cleveland Clinic. The other authors have no conflicts to declare.

## Supporting information



Supporting Information

## Data Availability

The data that support the findings of this study are available from the corresponding author upon reasonable request.

## References

[1] C. P. Blobel , “ADAMs: Key Components in EGFR Signalling and Development,” Nature Reviews Molecular Cell Biology 6, no. 1 (2005): 32–43, http://www.ncbi.nlm.nih.gov/entrez/query.fcgi?cmd=Retrieve&db=PubMed&dopt=Citatio&list_uids=15688065. 15688065 10.1038/nrm1548

[2] C. Bonnans , J. Chou , and Z. Werb , “Remodelling the Extracellular Matrix in Development and Disease,” Nature Reviews Molecular Cell Biology 15, no. 12 (2014): 786–801, 10.1038/nrm3904. 25415508 PMC4316204

[3] E. S. Radisky , “Extracellular Proteolysis in Cancer: Proteases, Substrates, and Mechanisms in Tumor Progression and Metastasis,” Journal of Biological Chemistry 300, no. 6 (2024): 107347, 10.1016/j.jbc.2024.107347. 38718867 PMC11170211

[4] N. Scamuffa , F. Calvo , M. Chrétien , N. G. Seidah , and A.‐.M. Khatib , “Proprotein Convertases: Lessons From Knockouts,” FASEB Journal 20, no. 12 (2006): 1954–1963, 10.1096/fj.05-5491rev. 17012247

[5] U. auf dem Keller and C. M. Overall , “CLIPPER: An Add‐on to the Trans‐Proteomic Pipeline for the Automated Analysis of TAILS N‐Terminomics Data,” Biological Chemistry 393, no. 12 (2012): 1477–1483, 10.1515/hsz-2012-0269. 23667905

[6] S. Bhutada , A. Hoyle , N. S. Piuzzi , and S. S. Apte , “Degradomics Defines Proteolysis Information Flow From Human Knee Osteoarthritis Cartilage to Matched Synovial Fluid and the Contributions of Secreted Proteases ADAMTS5, MMP13 and CMA1 to Articular Cartilage Breakdown,” Osteoarthritis and Cartilage 33, no. 1 (2025): 116–127, 10.1016/j.joca.2024.09.002. 39293776 PMC12342059

[7] S. Bhutada , L. Li , B. Willard , G. Muschler , N. Piuzzi , and S. S. Apte , “Forward and Reverse Degradomics Defines the Proteolytic Landscape of Human Knee Osteoarthritic Cartilage and the Role of the Serine Protease HtrA1,” Osteoarthritis and Cartilage 30, no. 8 (2022): 1091–1102, 10.1016/j.joca.2022.02.622. 35339693

[8] D. R. Martin , J. C. Witten , C. D. Tan , et al., “Proteomics Identifies a Convergent Innate Response to Infective Endocarditis and Extensive Proteolysis in Vegetation Components,” JCI Insight 5, no. 14 (2020), 10.1172/jci.insight.135317. PMC7453909 32544089

[9] A. Prudova , V. Gocheva , U. auf dem Keller , et al., “TAILS N‐Terminomics and Proteomics Show Protein Degradation Dominates Over Proteolytic Processing by Cathepsins in Pancreatic Tumors,” Cell Reports 16, no. 6 (2016): 1762–1773, 10.1016/j.celrep.2016.06.086. 27477282

[10] P. Kaushal and C. Lee , “N‐Terminomics—Its Past and Recent Advancements,” Journal of Proteomics 233, (2021): 104089, 10.1016/j.jprot.2020.104089. 33359939

[11] L. D. Rogers and C. M. Overall , “Proteolytic Post‐Translational Modification of Proteins: Proteomic Tools and Methodology,” Molecular & Cellular Proteomics 12, no. 12 (2013): 3532–3542, 10.1074/mcp.M113.031310. 23887885 PMC3861706

[12] O. Kleifeld , A. Doucet , U. auf dem Keller , et al., “Isotopic Labeling of Terminal Amines in Complex Samples Identifies Protein N‐Termini and Protease Cleavage Products,” Nature Biotechnology 28, no. 3 (2010): 281–288, 10.1038/nbt.1611. 20208520

[13] T. Kockmann , N. Carte , S. Melkko , and U. auf dem Keller , Identification of Protease Substrates in Complex Proteomes by iTRAQ‐TAILs on a Thermo Q Exactive Instrument, ed. J. E. Grant and H. Li (Springer Science+Business Media, 2016): 187–207.

[14] U. auf dem Keller , A. Prudova , U. Eckhard , B. Fingleton , and C. M. Overall , “Systems‐Level Analysis of Proteolytic Events in Increased Vascular Permeability and Complement Activation in Skin Inflammation,” Science Signaling 6, no. 258 (2013): rs2, 10.1126/scisignal.2003512. 23322905 PMC3872078

[15] A. Prudova , K. Serrano , U. Eckhard , N. Fortelny , D. V. Devine , and C. M. Overall , “TAILS N‐Terminomics of Human Platelets Reveals Pervasive Metalloproteinase‐Dependent Proteolytic Processing in Storage,” Blood 124, no. 26 (2014): e49–e60, 10.1182/blood-2014-04-569640. 25331112 PMC4271184

[16] P. Schlage , F. E. Egli , P. Nanni , et al., “Time‐Resolved Analysis of the Matrix Metalloproteinase 10 Substrate Degradome,” Molecular & Cellular Proteomics , 13, no. 2 (2014): 580–593, 10.1074/mcp.M113.035139. PMC3916655 24281761

[17] K. Kalogeropoulos , A. Moldt Haack , E. Madzharova , et al., “CLIPPER 2.0: Peptide‐Level Annotation and Data Analysis for Positional Proteomics,” Molecular & Cellular Proteomics 23, no. 6 (2024): 100781, 10.1016/j.mcpro.2024.100781. 38703894 PMC11192779

[18] A. J. Barrett , N. D. Rawlings , and E. A. O'brien , “The MEROPS Database as a Protease Information System,” Journal of Structural Biology 134, no. 2–3 (2001): 95–102, 10.1006/jsbi.2000.4332. 11551172

[19] N. Fortelny , S. Yang , P. Pavlidis , P. F. Lange , and C. M. Overall , “Proteome TopFIND 3.0 With TopFINDer and PathFINDer: Database and Analysis Tools for the Association of Protein Termini to Pre‐ and Post‐Translational Events,” Nucleic Acids Research 43, no. Database issue (2015): D290–D297, 10.1093/nar/gku1012. 25332401 PMC4383881

[20] P. F. Lange , P. F. Huesgen , and C. M. Overall , “TopFIND 2.0–linking Protein Termini with Proteolytic Processing and Modifications Altering Protein Function,” Nucleic Acids Research 40, no. Database issue (2012): D351–D361, 10.1093/nar/gkr1025. 22102574 PMC3244998

[21] M. Rydén , A. Turkiewicz , P. Önnerfjord , J. Tjörnstrand , M. Englund , and N. Ali , “Identification and Quantification of Degradome Components in Human Synovial Fluid Reveals an Increased Proteolytic Activity in Knee Osteoarthritis Patients Vs Controls,” Proteomics 23, no. 15 (2023): 2300040, 10.1002/pmic.202300040. 37226369

[22] S. S. Apte and W. C. Parks , “Metalloproteinases: A Parade of Functions in Matrix Biology and an Outlook for the Future,” Matrix Biology 44–46 (2015): 1–6, 10.1016/j.matbio.2015.04.005. 25916966

[23] H. Wang and O. Julien , “CaspSites: A Database and Web Application for Experimentally Observed Human Caspase Substrates Using N‐Terminomics,” Journal of Proteome Research 22, no. 2 (2023): 454–461, 10.1021/acs.jproteome.2c00620. 36696595

[24] R. Dobrota , S. Jordan , P. Juhl , et al., “Circulating Collagen Neo‐Epitopes and Their Role in the Prediction of Fibrosis in Patients With Systemic Sclerosis: A Multicentre Cohort Study,” Lancet Rheumatology 3, no. 3 (2021): e175–e184, 10.1016/s2665-9913(20)30385-4. 38279380

[25] C. E. Hughes , B. Caterson , A. J. Fosang , P. J. Roughley , and J. S. Mort , “Monoclonal Antibodies That Specifically Recognize Neoepitope Sequences Generated by ‘Aggrecanase’ and Matrix Metalloproteinase Cleavage of Aggrecan: Application to Catabolism In Situ and In Vitro,” Biochemical Journal 305, no. pt. 3 (1995): 799–804, http://www.ncbi.nlm.nih.gov/pubmed/7531436. 7531436 10.1042/bj3050799 PMC1136329

[26] J. S. Mort , C. R. Flannery , J. Makkerh , J. C. Krupa , and E. R. Lee , “Use of Anti‐Neoepitope Antibodies for the Analysis of Degradative Events in Cartilage and the Molecular Basis for Neoepitope Specificity,” Biochemical Society Symposia , no. 70 (2003): 107–114, http://www.ncbi.nlm.nih.gov/entrez/query.fcgi?cmd=Retrieve&db=PubMed&dopt=Citation&list_uids=14587286. 10.1042/bss0700107 14587286

[27] J. D. Sandy , J. Westling , R. D. Kenagy , et al., “Versican V1 Proteolysis in Human Aorta in Vivo Occurs at the Glu441‐ Ala442 Bond, a Site That Is Cleaved by Recombinant ADAMTS‐1 and ADAMTS‐ 4,” Journal of Biological Chemistry 276, no. 16 (2001): 13372–13378, http://www.ncbi.nlm.nih.gov/htbin‐post/Entrez/query?db=m&form=6&dopt=r&uid=11278559, http://www.jbc.org/cgi/content/full/276/16/13372, http://www.jbc.org/cgi/content/abstract/276/16/13372. 11278559 10.1074/jbc.M009737200

[28] S. Santamaria and R. De Groot , “ADAMTS Proteases in Cardiovascular Physiology and Disease,” Open Biology 10, no. 12 (2020): 200333, 10.1098/rsob.200333. 33352066 PMC7776578

[29] F. Desiere , E. W. Deutsch , N. L. King , et al., “The PeptideAtlas Project,” Nucleic Acids Research 34 no. Database issue (2006): D655–D658, 10.1093/nar/gkj040. 16381952 PMC1347403

