# Supplementary material for: DICED (Database of Identified Cleavage Sites Endemic to Diseases States): A Searchable Web Interface for Terminomics/Degradomics
Source: Proteomics. 2025 May 12;25(13):e202500007. doi: 10.1002/pmic.202500007 (PMC12246766; doi:10.1002/pmic.202500007)
Supplement: Supplementary file 1 — Supporting Information [file PMIC-25-e202500007-s001.pdf]

Manuscript supporting information

Technical Brief

DICED (Database of identified cleavage sites endemic to diseases states): A searchable web interface for terminomics

Jayadev Joshi, Sumit Bhutada, Daniel R. Martin, Daniel Blankenberg & Suneel S. Apte

The supplement contains Figures S1-S4

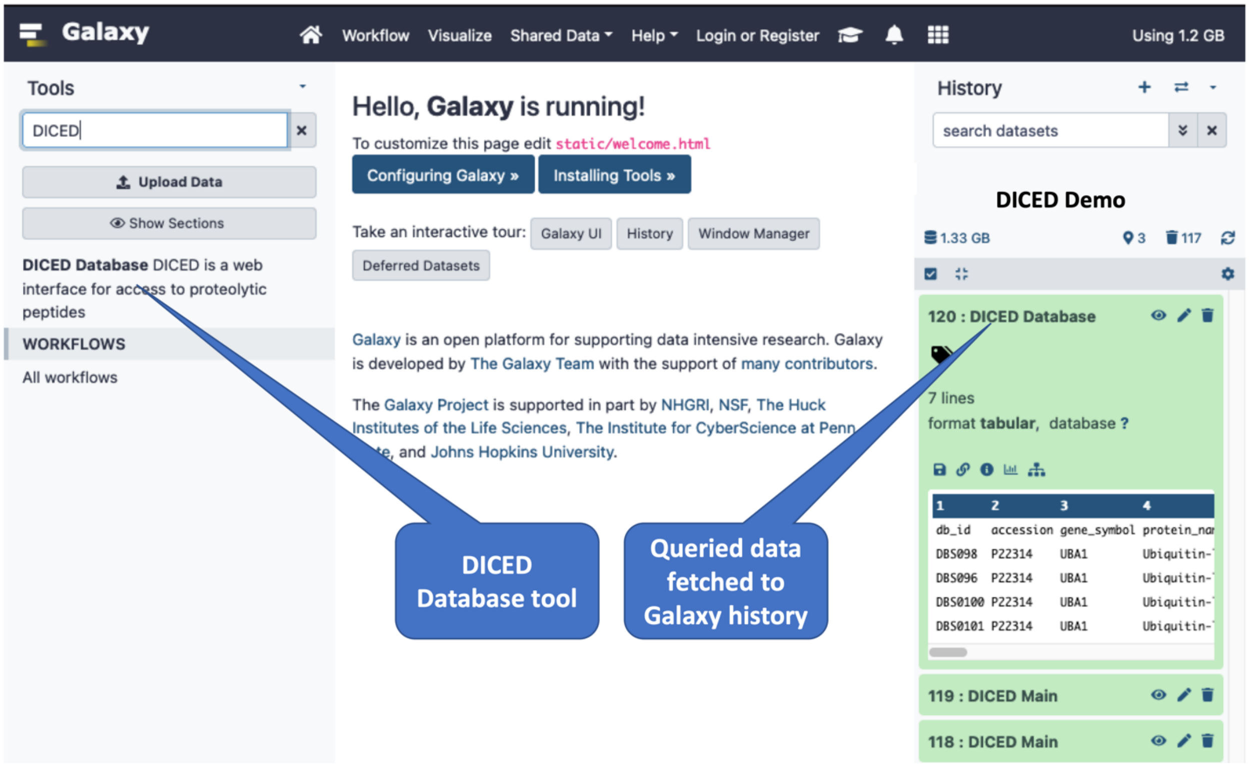

Figure S1: **DICED, a Galaxy data-source tool:** This tool allows users to fetch query output data directly within the Galaxy interface.

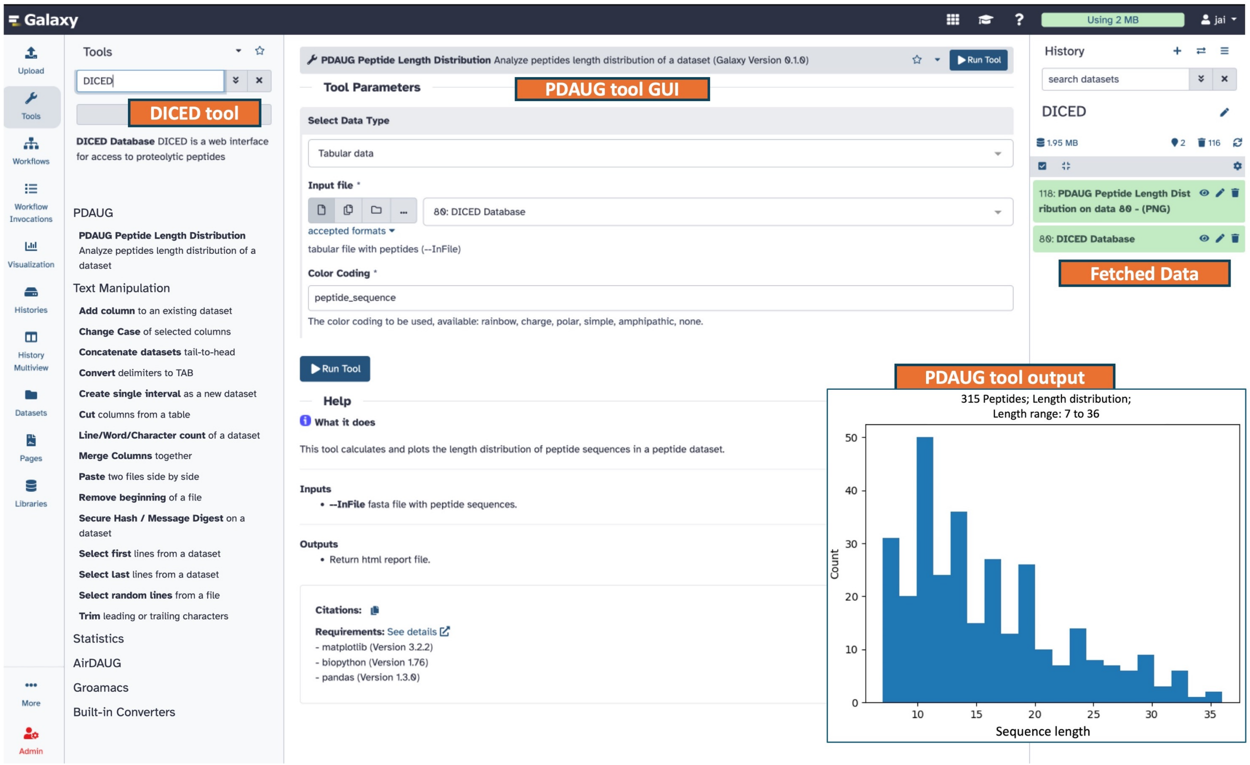

Figure S2: DICED integration with Galaxy. In the illustrated example, the left-hand menu lists DICED and other tools. The central section (PDAUG tool GUI) shows the “PDAUG Peptide Length Distribution” tool. The right-hand history panel (Fetched Data) presents the data fetched from the DICED server, with the top dataset showing the length distribution. The resulting distribution histogram is displayed in the bottom right-hand corner.

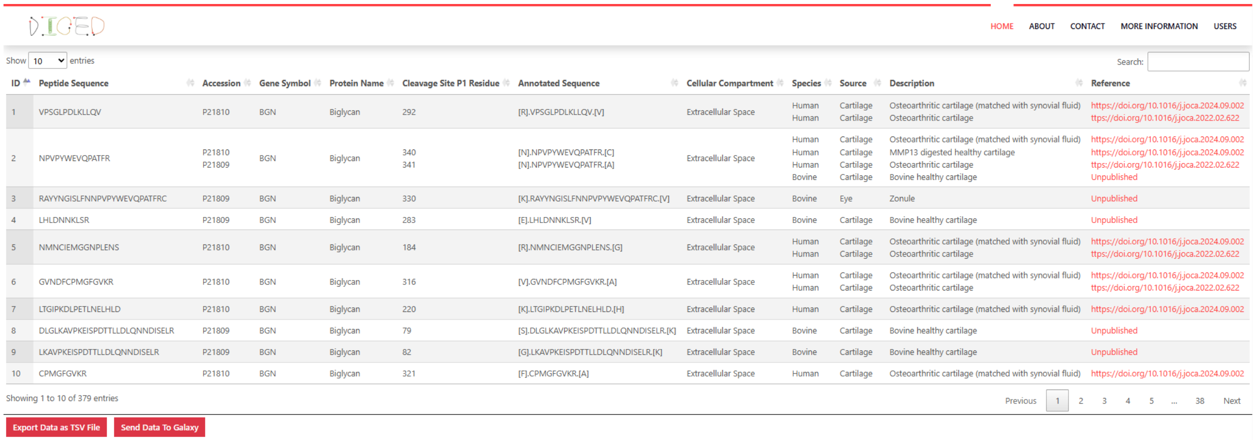

HOME ABOUT CONTACT MORE INFORMATION USERS

Show 10 entries

Search:

ID	Peptide Sequence	Accession	Gene Symbol	Protein Name	Cleavage Site P1 Residue	Annotated Sequence	Cellular Compartment	Species	Source	Description	Reference
1	VPSGLPDLKLLQV	P21810	BGN	Biglycan	292	[R].VPSGLPDLKLLQV.[V]	Extracellular Space	Human<br>Human	Cartilage<br>Cartilage	Osteoarthritic cartilage (matched with synovial fluid)<br>Osteoarthritic cartilage	https://doi.org/10.1016/j.joca.2024.09.002<br>ttps://doi.org/10.1016/j.joca.2022.02.622
2	NPVPYWEVQPATFR	P21810<br>P21809	BGN	Biglycan	340<br>341	[N].NPVPYWEVQPATFR.[C]<br>[N].NPVPYWEVQPATFR.[A]	Extracellular Space	Human<br>Human<br>Human<br>Bovine	Cartilage<br>Cartilage<br>Cartilage<br>Cartilage	Osteoarthritic cartilage (matched with synovial fluid)<br>MMP13 digested healthy cartilage<br>Osteoarthritic cartilage<br>Bovine healthy cartilage	https://doi.org/10.1016/j.joca.2024.09.002<br>https://doi.org/10.1016/j.joca.2024.09.002<br>ttps://doi.org/10.1016/j.joca.2022.02.622<br>Unpublished
3	RAYYNGISLFNNPVPYWEVQPATFRC	P21809	BGN	Biglycan	330	[K].RAYYNGISLFNNPVPYWEVQPATFRC.[V]	Extracellular Space	Bovine	Eye	Zonule	Unpublished
4	LHLDNNKLSR	P21809	BGN	Biglycan	283	[E].LHLDNNKLSR.[V]	Extracellular Space	Bovine	Cartilage	Bovine healthy cartilage	Unpublished
5	NMNCIEMGGNPLENS	P21810	BGN	Biglycan	184	[R].NMNCIEMGGNPLENS.[G]	Extracellular Space	Human<br>Human	Cartilage<br>Cartilage	Osteoarthritic cartilage (matched with synovial fluid)<br>Osteoarthritic cartilage	https://doi.org/10.1016/j.joca.2024.09.002<br>ttps://doi.org/10.1016/j.joca.2022.02.622
6	GVNDFCPMGFGVKR	P21810	BGN	Biglycan	316	[V].GVNDFCPMGFGVKR.[A]	Extracellular Space	Human<br>Human	Cartilage<br>Cartilage	Osteoarthritic cartilage (matched with synovial fluid)<br>Osteoarthritic cartilage	https://doi.org/10.1016/j.joca.2024.09.002<br>ttps://doi.org/10.1016/j.joca.2022.02.622
7	LTGIPKDLPETLNELHLD	P21810	BGN	Biglycan	220	[K].LTGIPKDLPETLNELHLD.[H]	Extracellular Space	Human	Cartilage	Osteoarthritic cartilage (matched with synovial fluid)	https://doi.org/10.1016/j.joca.2024.09.002
8	DLGLKAVPKEISPDTTLLDLQNNDISELR	P21809	BGN	Biglycan	79	[S].DLGLKAVPKEISPDTTLLDLQNNDISELR.[K]	Extracellular Space	Bovine	Cartilage	Bovine healthy cartilage	Unpublished
9	LKAVPKEISPDTTLLDLQNNDISELR	P21809	BGN	Biglycan	82	[G].LKAVPKEISPDTTLLDLQNNDISELR.[K]	Extracellular Space	Bovine	Cartilage	Bovine healthy cartilage	Unpublished
10	CPMGFGVKR	P21810	BGN	Biglycan	321	[F].CPMGFGVKR.[A]	Extracellular Space	Human	Cartilage	Osteoarthritic cartilage (matched with synovial fluid)	https://doi.org/10.1016/j.joca.2024.09.002

Showing 1 to 10 of 379 entries

Previous 1 2 3 4 5 ... 38 Next

Export Data as TSV File Send Data To Galaxy

Figure S3: Data output page from a search using the protein name “biglycan”, showing retrieval of peptides from human and bovine N-terminomes and reverse degradomes. For example, the peptide in row 2 was detected in human osteoarthritis cartilage, healthy bovine cartilage and the MMP13 degradome. The search additionally reveals peptides found in cartilage and ocular zonule.

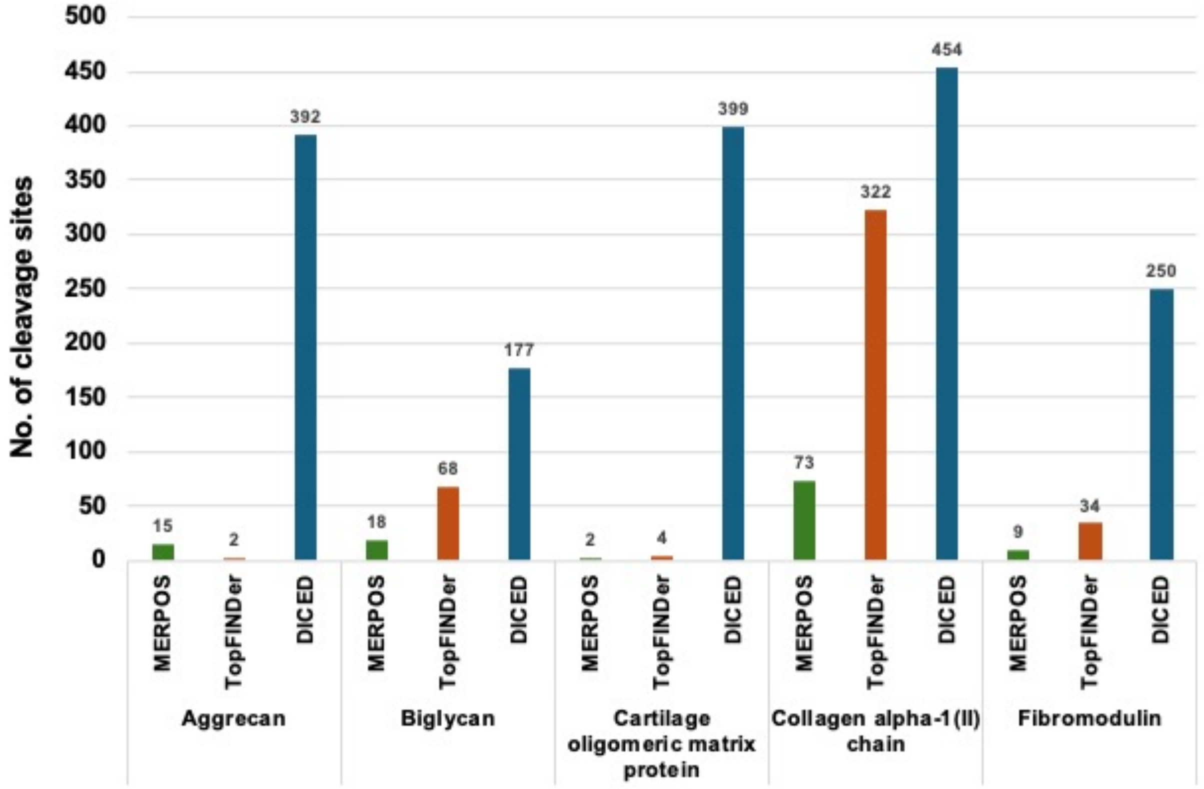

Figure S4. Comparison of output from TopFINDer, MEROPS and DICED. Searches were done for the 5 proteins shown, with the greatest peptide yield for each appearing in DICED. The data illustrates that the majority of cleavages in the DICED output, which were not found in either MEROPS or TopFINDer, result from the activities of unknown proteases.
